# The Role of Acute Trigemino-Cardiac Reflex in Unusual, Non-Surgical Cases: A Review

**DOI:** 10.3389/fneur.2016.00186

**Published:** 2016-10-26

**Authors:** Tumul Chowdhury, Bernhard Schaller

**Affiliations:** ^1^Department of Anesthesiology and Perioperative Medicine, University of Manitoba, Winnipeg, MB, Canada; ^2^Department of Research, University of Southampton, Southampton, UK

**Keywords:** trigemino-cardiac reflex, asystole, death, brainstem reflex

## Abstract

Trigemino-cardiac reflex (TCR) is a well-established phenomenon that is mainly reported in the various surgical specialties. However, the role of this unique reflex is entirely unknown in other medicine domains. Therefore, the present mini-review aims to explore the role of TCR in such unusual cases and also highlights the importance of case reports for knowledge creation in such context.

## Introduction

In the last 20 years, there has been a substantial interest among the neuroscientists about the unique brainstem reflex, known as “trigemino-cardiac reflex” (TCR). This reflex is usually incited by the stimulation of the fifth nerve along its entire course, anywhere from the peripheral division to the central connections (1–5). Interestingly, the occurrence of the TCR episodes is not only limited to neurosurgical procedures/interventions but also explored and reported in various other non-neurosurgical procedures including ocular surgeries, oral–facial–maxillary surgeries, dental procedures, dermatological surgeries, etc. (1–12). In general, irrespective of the nature of the surgery, the common manifestation of the TCR remains almost similar that include bradycardia, hypotension, apnea, and/or gastric hypermotility. In addition to the usual association of the TCR to various surgical procedures in the vicinity of the trigeminal nerve, there are only very few reports that do highlight the unusual as well as the catastrophic nature of the TCR in non-surgical conditions, and further expands the knowledge and understanding of TCR in other domains of medicine. Such reports underline the connection from the TCR to the dive reflex or sudden infant death syndrome. From the extensive surgical/interventional reports, however, one might assume that such non-surgical cases might be underreported. Therefore, the present mini-review aims to explore the role of TCR in such unusual cases and also highlights the importance of case reports for knowledge creation in such context.

## Method

### Definition of the TCR

The TCR is defined here as the sudden onset of parasympathetic dysrhythmia, hypotension, apnea, or gastric hypermotility during stimulation of any of the sensory branches of the trigeminal nerve. A TCR should include a decrease in heart rate (HR) and mean arterial blood pressure (MABP) of more than 20% as compared with baseline values before application of the stimulus and coincide with the surgical manipulation at or around any branches of the trigeminal nerve (7).

### Review of Literature

We have searched terms including “trigemino-cardiac reflex,” “trigeminal-cardiac reflex,” “oculocardiac reflex (OCR)” in various search engines including PubMed, Google, EMBASE, and SCOPUS from January 1, 1970 to August 1, 2016. All cross references are also carefully reviewed.

#### Inclusion Criteria

Articles that clearly described TCR or OCR as a cause of hemodynamic/respiratory changes as defined above in any age group and either of the sexes in human subjects was included. All the papers irrespective of the type and language were also included in this review (Table 1). Episodes of TCR or OCR that were followed by surgery or other neuro-interventions were also included.

**Table 1:** **Unusual cases of trigemino-cardiac reflex**.

S. no.	Age/gender	Procedure	Hemodynamics	Outcome
1	65/M	Nasal packing	Bradycardia, apnea, hypotension	Died
2	69/M	Nasal packing	Dyspnea, bradycardia, pulseless electrical activity	Died
3	58/M	Suicidal fire	Cardiac arrest	Died
4	Elderly/F	Homicide	Cardiac arrest	Died

#### Exclusion Criteria

Patients with prior history of cardiac disease, b-blockers, other medications prone to cause bradycardia, and TCR or OCR episodes occurring during any surgical or interventional procedures were excluded. Chronic TCR appearance was excluded. Injury or inciting events that predisposed the TCR or the OCR episodes and reported in three or more than three papers were also excluded.

## Results

Out of all the screened papers, only four articles met the stringent inclusion and exclusion criteria (13–16). Out of these four, two cases (50%) report epistaxis and two (50%) forensic investigations (Table 1). All the four patients died because of cardiac arrest. Out of the four patients mentioned in these reports, three (75%) were male of 56–69 years of age and one (25%) was elderly (age not mentioned) female.

First two papers highlighted that epistaxis was a presenting symptom, and the nasal packing was done to control the nasal bleeding in both the patients (13, 14). After the nasal packing, the first patient (65/M) developed bradycardia, a decrease in oxygen saturation (50%), and hypotension after 15 min, whereas the second patient (69/M) first showed respiratory distress (oxygen saturation 76%) followed by bradycardia. Both the patients were managed by endotracheal intubation and advanced cardiac life support (ACLS). Unfortunately, both the patients could not be revived back and died. Strikingly, nasal packs were not removed during the resuscitation phase in both the patients. Other two papers reported the forensic involvement (15, 16). Here, the first forensic report, a 58-year-old male with a history of depression presented with a suicidal fire case. Interestingly, in the first instance, the external examination and other forensic evaluation reports directed the cause of death as one of the mechanisms related to burn; however, in the absence of obvious pathological findings of burn, subsequent internal examination, and toxicological analysis ruled out the usual mechanisms and postulated the neurogenic mechanism (TCR). The second case, a violent stabbing on the elderly female led to bilateral ocular injuries and multiple facial and neck injuries. Interestingly, at the first instance, it appeared that the cause of death was head and neck injuries. However, subsequent investigations revealed no obvious cause and thus suspected OCR (a peripheral TCR) as the cause of this demise.

## Discussion

Trigemino-cardiac reflex is an interesting phenomenon that hitherto has primarily been focused on different surgical procedures including neurosurgical/neuro-interventions, dental, oral–facial–maxillary, and ocular surgeries in which upon the stimulation of the trigeminal nerve along its entire course predisposes the cardiorespiratory and gastric symptoms. These include negative chronotropic from mild (bradycardia) to severe (asystole) changes, bradypnea, apnea, and gastric hypermotility (1–12). Although the mechanism of the TCR is very complex but generally accepted mechanism includes that following the stimulation of the any sensory branch of the trigeminal nerve, signals are carried to the sensory nucleus of the trigeminal nerve *via* the Gasserian ganglion. This afferent pathway continues along the short internuncial nerve fibers in the reticular formation to connect with the efferent pathway in the motor nucleus of the vagus nerve. These terminate in the cardiac ganglia from which the postganglionic fibers are sent to the conductive system, leading to autonomic changes that usually manifest as a negative chronotropy (5, 17). So far, extensive investigations have been done to extract the role of TCR in various surgical procedures, neuro-interventions, different manifestations (cardiorespiratory changes), various definitions, and effect on outcome; however, the TCR in the majority of the reported literature seems a mild manifestation that is usually transient in nature (1–12).

Strikingly, after reviewing these above four papers, it seems clear that the TCR phenomenon is not merely limited to the surgical domain. Instead, it can have deleterious effects in other non-surgical specialties or procedures. The two cases of nasal packing induced severe cardiorespiratory disturbances clearly highlighted that the TCR episodes could result in fatal outcome. It worth to be mentioned that a fatal outcome is rarely described during surgery/intervention; if this is a publication bias or a characteristic phenomenon of the non-surgical cases. Notably, the nasal packs in both the cases were not removed during these events (13, 14). Whether or not, the outcome could have been different if these nasal packs would have been taken out is a matter of further research in animal models. However, as per the classical definition of the TCR, the removal of the nasal pack could have been aborted the TCR and related deleterious effect. These two cases certainly show the paramount importance of knowledge and recognition of TCR episodes in such a common scenario. On the other hand, the two other papers related to forensic pathology did reveal the importance of this unique brain stem reflex for the scenarios in which there were lack substantial evidence or clues. In the homicidal fire case, the thermal stimulation on the face incited the TCR and thus patient sudden cardiac arrest (15). Similarly, in the second case, the penetrating injuries in eyes and orbits triggered the OCR, a peripheral subtype of TCR and resulted in the sudden death of the patient (16). Whether or not, sudden death in such cases (where forensic investigations, autopsy, and toxicological analysis do not reveal apparent cause) could be due to TCR/OCR is a matter of further research.

In all four cases, there was a clear cause-and-effect relationship. On the one side, this is evidence of a TCR, and the stimulation of the trigeminal root, on the other hand, this represents as well as confirms the afferent pathway of the TCR. If just one such observational case does not fit completely with the initial proposition, the hypothesis should be considered generally not valid and therefore should be revised or even rejected. This was not the case with our hypothesis of the existence of the TCR in non-surgical case reports and demonstrates that – besides the qualitative method of triangulation – the ongoing description of similar cases about a specific topic has importance not only for clinical practice but also for the scientific community (18, 19). However, after reviewing these non-surgical cases, the TCR phenomenon would certainly add the list of differential diagnoses in such situations. Also, this review also justifies that the case reports can also provide substantial knowledge and understanding of such phenomenon like the TCR. Our previous work also supports this notion that the case reports can have extraordinary significance for the elucidation of the rarer yet clinically relevant phenomenon, such as TCR.

Our work has some limitations. We have only found four cases, so that this case series does not impart the complete and more specific knowledge of TCR in such cases. However, the trend is so strong (hundreds of surgical cases without any fatal outcome and four of the four cases with fatal outcome) that we should think about an adaption of our previous model of the TCR. This is also the second principal limitation that we cannot yet explain this differential behavior in a meta-context.

In conclusion, this review shows that the existence of the TCR episodes can also occur in non-surgical as well as simple maneuvers and can lead to devastating complications including death. Therefore, this review further opens the understanding of the TCR phenomenon in various medicine domains as well.

## Author Contributions

TC helped in developing the concept, designing, data collection, data interpretation, and writing the manuscript. BS gave substantial inputs in developing the concept and writing the manuscript. Both the authors accepted the final version.

## Conflict of Interest Statement

The authors declare that the research was conducted in the absence of any commercial or financial relationships that could be construed as a potential conflict of interest.
